# "Medical Aid" and "Friendly" Societies

**Published:** 1899-06-17

**Authors:** 


### The Hospital

A Journal of

The Medical Sciences and Hospital Administration.

Vol. XXVI.—No. 664.] Edited by Sir Henry Burdett, K.C.B., and Solomon C. Smith, M.D., M.R.C.P. [June 17, 1899.

### "Medical Aid" and "Friendly" Societies.

Among the questions which, during the last few years, have from time to time occupied the attention of the General Medical Council, few are more important to large numbers of people than the establishment or maintenance of correct relations between the members of the medical profession and the various societies and associations which contract with doctors for attendance in time of sickness, in return for small regular payments, made independently of its occurrence. The societies in question may be roughly divided into three classes: The genuine friendly societies, registered under a special Act of Parliament, and giving to their members not only medical attendance, but a weekly payment during illness and a contribution towards the expenses of a funeral; the "medical aid" associations or so-called "provident dispensaries," which deal with medical attendance only; and the many modern insurance companies, which contract with a doctor for the supply of such attendance to their customers, and throw in his services as a benefit to be obtained along with the policy. It is obvious that, under all three of these forms, it would be possible to make mutually advantageous arrangements; but it has also been shown by experience that all of them are liable to give rise to serious abuses. A doctor who undertakes to attend the members of a friendly society, in return for a payment from each of them of a few shillings a year, rightly thinks that he is rendering them very valuable assistance towards the maintenance of self-respect and independence, and reasonably objects to do for comparatively wealthy people what he is perfectly willing to do for a genuine "working man." But it has happened in many instances that the societies have contained members who were in a position superior to that of a working man at the time of joining, or who had risen into a superior position in course of time. Such members are often of great value to the society itself, bringing shrewdness, knowledge of business, and power of organisation to assist in the management of its affairs; but they are well able to pay for medical attendance at ordinary rates, their very prosperity is sometimes conducive to habits less favourable to health than those of the veritable wage-earner, and it is not just that the doctor should find them among the number of his contract patients. Many of these men feel this themselves, and voluntarily relinquish medical benefits; but others cling with tenacity to what they regard as their right, and often think themselves neglected unless they receive more attention in illness than would be either wished for by, or given to, an ordinary member. When the wage-earning member would be willing to attend at the doctor's house, the wealthy member expects to be visited at his own, although a legitimate charge for a single visit would be as much as he pays during the year. Friction arising from this and similar causes has in many places produced lamentable differences between the doctors and the friendly societies. The latter have, in some instances, brought new doctors into the town as their salaried servants, have set up dispensaries and engaged dispensers, and have then striven to extend their membership as much as possible, to the great detriment of the practitioners previously established. The latter have appealed to the Medical Council, in the hope that the conduct of the imported rivals might be declared "infamous in a professional respect," and that they might be removed from the Register. A moment's reflection would show this to be impossible; but the Council appointed a committee to investigate the facts, and to confer with representatives of the friendly societies. Several meetings have been held, with the result, we hope, of showing the comparative unimportance of many of the points of difference, and the great advantages, both to the societies and to the medical profession, which would follow from an amicable settlement of all the questions at issue. To this end the conference has recommended the formation of a board of conciliation to which disputes between the societies and their doctors may be referred, and thus lifted out of the region of personal controversy, to be settled on some basis of principle. The representatives of the societies have agreed to urge the adoption of this course upon their constituents, and the Medical Council has approved it by resolution. With regard to the two other forms of "medical aid" there are still many difficulties in the way of a satisfactory solution of the problems which they present, not the least being to find a method of providing, at a rate within the means of the poor, medical attendance which shall really be worth even the modest sum which those means are capable of providing. The "advice and medicine" of a cheap "dispensary" may easily bear, to sound advice and veritable remedies, much the same relation that was borne by the Irishman's sedan chair to his more accustomed method of locomotion. The chair had no seat and no bottom, and, "but for the name of the thing," he might as well have walked.

